# Genome-Based Reclassification of *Streptococcus taoyuanensis* ST2^T^ as a Later Heterotypic Synonym of *Streptococcus caecimuris* CLA-AV-18^T^

**DOI:** 10.3390/microorganisms14040766

**Published:** 2026-03-27

**Authors:** Fangqiu Ding, Tong Wang, Ruimeng Sun, Yuli Wei, Yong Wu, Miao Yu, Yuguo Tang

**Affiliations:** 1 School of Biomedical Engineering (Suzhou), Division of Life Sciences and Medicine, University of Science and Technology of China, Hefei 230026, China; dingfq@sibet.ac.cn; 2 CAS Key Laboratory of Bio-Medical Diagnostics, Suzhou Institute of Biomedical Engineering and Technology, Chinese Academy of Sciences, Suzhou 215163, China; 3 Center for Life Science, School of Life Sciences, Yunnan University, Kunming 650500, China; tong.wang@acxel.com; 4 Department of Biomaterials and Stem Cells, Suzhou Institute of Biomedical Engineering and Technology, Chinese Academy of Sciences, Suzhou 215163, China; sunruimeng0507@163.com; 5 Shanghai Engineering Research Center of Hadal Science and Technology, College of Oceanography and Ecological Science, Shanghai Ocean University, Shanghai 201306, China; yl_wei@shou.edu.cn (Y.W.); yong.wu@acxel.com (Y.W.)

**Keywords:** *Streptococcus taoyuanensis*, *Streptococcus caecimuris*, genome-based reclassification, heterotypic synonym

## Abstract

This study systematically evaluated the taxonomic relationship between *Streptococcus taoyuanensis* ST2^T^ and *Streptococcus caecimuris* CLAAV18^T^. Comparative genomic analysis revealed a high 16S rRNA gene sequence similarity of 99.6%, with the two strains clustering closely in the 16S rRNA-based phylogenetic tree. The genetic relatedness was further validated by Multi-Locus Sequence Typing (MLST) analysis: assessments of seven conserved housekeeping genes (*atpD*, *gapA*, *gyrB*, *GdhA*, *recA*, *dnaK*, and *sdhA*) demonstrated complete concordance in target fragment lengths (ranging from 33 bp to 121 bp). No size polymorphisms, insertions, or deletions were detected, indicating a highly conserved core genome. At the whole-genome level, the Average Amino Acid Identity (AAI), Average Nucleotide Identity (ANI), and digital DNA-DNA hybridization (dDDH) values between the two strains were 96.8%, 95.7%, and 84.6%, respectively. These values significantly exceed the established thresholds for species delineation (AAI: 95.5%; ANI: 95%; dDDH: 70%), providing robust genomic evidence that both strains belong to the same species. Furthermore, phenotypic testing confirmed nearly identical physiological characteristics, with only minor biochemical variations. Based on the integration of phylogenetic, genomic, and phenotypic evidence, we formally propose *Streptococcus taoyuanensis* as a later heterotypic synonym of *Streptococcus caecimuris*.

## 1. Introduction

The genus *Streptococcus* is a member of the family *Streptococcaceae* with an increasing number of new species described in recent years. The genus was first proposed by Rosenbach [1], and its taxonomic history has been comprehensively reviewed by Wilson & Miles [2] and Jones [3]. The genus *Streptococcus* comprises 140 species with validly published names (https://lpsn.dsmz.de/genus/Streptococcus, accessed on 24 February 2026 [4]). *Streptococcus* has played an important role in clinical infectious diseases, as some Streptococci are associated with endometritis, respiratory infections, endocarditis, meningitis, arthritis and mastitis. The majority of species are pathogens or commensals in humans and animals, including significant veterinary pathogens, such as *Streptococcus suis* in pigs [5], *Streptococcus uberis* in bovines [6] and *Streptococcus equi* subsp. *equi* in horses [7]. All members belonging to this genus are Gram-stain-positive cocci, which form pairs or chains, catalase-negative and facultative anaerobes. *S. caecimuris* CLA-AV-18^T^ (=DSM 110150^T^ =JCM 36502^T^) was isolated from the caecum of a mouse [8] and was validated in the International Journal of Systematic and Evolutionary Microbiology (IJSEM) (Validation List No. 216) [9]. *Streptococcus taoyuanensis*, with the type strain ST2^T^ (=BCRC 81374^T^ =NBRC 115928^T^), was isolated from a patient with bacteremia by Lee et al. [10] and was validated in IJSEM (Validation List No. 224) [11]. The two strains shared a 99.6% 16S rRNA gene sequence similarity, indicating that they might represent the same species. The taxonomic relationship between these two type strains was re-evaluated in this study.

Nowadays, modern phylogenetic approaches based on whole-genome sequencing have become essential for the taxonomic classification of prokaryotes [12,13]. Phylogenomic analysis based on the core genome, combined with overall genomic relatedness indices (OGRIs) including average nucleotide identity (ANI), digital DNA–DNA hybridization (dDDH), and average amino acid identity (AAI) [14,15], provides a more thorough and accurate insight into genetic relatedness. Such robust resolution cannot be attained using phenotypic assays, chemotaxonomic methods, or single-gene phylogenetic markers such as the 16S rRNA gene. These advanced genomic strategies have been extensively adopted for the reclassification of many bacterial taxa, enabling more accurate and evolutionarily consistent taxonomic assignments [16,17].

The priority of prokaryotic names is governed by the International Code of Nomenclature of Bacteria [18]. Rules 23b, 24a and 24b establish the priority of names based on their dates of valid publication in IJSEM. In this case, *S. caecimuris* was validly published on 28 March 2024 [9] and *S. taoyuanensis* was validly published on 31 July 2025 [11]. Based on the rules of priority, *S. taoyuanensis* is a later heterotypic synonym of *S. caecimuris*.

The taxonomic classification of prokaryotes serves as the foundation of microbiological research, and accurate species delineation is critical for studies on microbial diversity, pathogenicity, ecological adaptation, and public health surveillance. *Streptococcus* is a large and clinically important bacterial genus, and the continuous increase in newly described species has led to frequent nomenclatural confusion and redundant classification. In this study, we consider that *Streptococcus taoyuanensis* ST2^T^ and *Streptococcus caecimuris* CLAAV18^T^ were proposed as two separate species in different publications, but their taxonomic relationship had not been rigorously verified.

## 2. Materials and Methods

### 2.1. Genomic Dataset

The 16S rRNA gene sequence similarity between *S. taoyuanensis* ST2^T^ and *S. caecimuris* CLA-AV-18^T^ was determined using the pairwise alignment tool available on the EzBioCloud server (https://www.ezbiocloud.net/tools/pairAlign, accessed on 24 October 2025). The 16S rRNA gene sequences were retrieved from the genomes of strains ST2^T^ and CLA-AV-18^T^ via the BLAST program provided by the National Center for Biotechnology Information (https://www.ncbi.nlm.nih.gov/). The 16S rRNA gene sequences of closely related type strains were obtained from the EzBioCloud database (https://www.ezbiocloud.net/) [19]. Genome sequences and associated metadata of the strains investigated in this study were acquired from the NCBI Genome Portal (https://www.ncbi.nlm.nih.gov/datasets/genome/?taxon=1873, accessed on 24 October 2025). To ensure high-quality genomic data, the completeness and potential contamination of the recovered genome assemblies were evaluated using the CheckM software package [20].

### 2.2. Phylogenetic Analysis

Phylogenetic trees were reconstructed using the maximum-likelihood (ML) statistical method employing MEGA-X [21], a widely used and reliable software platform for molecular evolutionary analysis that offers robust tools for tree inference. The maximum-likelihood trees were reconstructed using Kimura’s two-parameter model, which is particularly suitable for analyzing nucleotide sequence data by accounting for different rates of transitions and transversions, along with transitions + transversions, uniform rates and complete deletion options. Bootstrap values based on 1000 replicates [22] were calculated to assess the reliability of each node in the phylogenetic trees, ensuring the statistical support for the inferred evolutionary relationships and enhancing the credibility of the observed phylogenetic clustering.

### 2.3. Multi-Locus Sequence Typing (MLST) Analysis

To validate the genetic relatedness between *S. taoyuanensis* ST2^T^ and *S. caecimuris* CLAAV18^T^, the Multilocus Sequence Typing (MLST) database was used as the core tool for sequence comparison, with seven conserved housekeeping genes (*atpD*, *gapA*, *gyrB*, *GdhA*, *recA*, *dnaK*, *sdhA*) selected as the target loci. First, the nucleotide sequences of the seven housekeeping genes from both strains were retrieved and trimmed to obtain high-quality target fragments. Subsequently, the trimmed sequences of each housekeeping gene from the two strains were separately submitted to the MLST database for alignment and comparison. The database was used to query and confirm the target fragment lengths of each gene, and the alignment results were analyzed to detect potential size polymorphisms, length variations, deletions, or insertions in the gene loci. The consistency of target fragment lengths between the two strains was determined based on the MLST database comparison results, so as to evaluate the genetic relatedness of the two strains at the housekeeping gene level.

### 2.4. Phylogenomic and Overall Genome Relatedness Indices

The draft genome sequences of *S. taoyuanensis* ST2^T^ (JARQVW000000000) and *S. caecimuris* CLA-AV-18^T^ (CALPCT010000000) were retrieved from the NCBI database. For phylogenomic investigation, the obtained genome datasets were submitted to the Type (Strain) Genome Server (TYGS; https://tygs.dsmz.de) [23], which includes the latest updates and improved functions described by Meier-Kolthoff et al. [24].

The average amino acid identity (AAI) was measured with an online calculator (http://enve-omics.ce.gatech.edu/aai, accessed on 24 October 2025) which adopts best and reciprocal best hits for accurate amino acid identity estimation between genomic protein datasets with strict alignment filters for reliable results. The average nucleotide identity (ANI) between two genomes was calculated with pairwise genome alignment using the ANI-BLAST (ANIb) and ANI-MUMmer (ANIm) algorithms implemented within the JSpeciesWS web service as described by Richter & Rosselló-Móra [14]. The digital DNA–DNA hybridization (dDDH) estimate value was analyzed using the genome-to-genome distance calculator (GGDC) [24,25,26], with contig files uploaded to the GGDC 3.0 Web server (https://ggdc.dsmz.de/ggdc.php#, accessed on 24 October 2025) for professional dDDH value calculations via its dedicated analytical pipeline.

### 2.5. Pangenomic Analysis

To assess genomic structure and variations within the two strains and closely related *Streptococcus* species, the Integrated Prokaryotes Genome and Pan-genome Analysis (IPGA) web server (https://nmdc.cn/ipga, accessed on 24 October 2025) [27] was utilized for systematic genomic and pan-genomic analyses. As a one-stop, tool-free online service for prokaryotic genome research, it supports comprehensive analysis including quality control, ANI calculation, pan-genome profiling and synteny analysis, and features a score system to evaluate the reliability of pan-genome profiles generated by different analytical packages, enabling the selection of the most suitable method for the dataset to ensure the accuracy of subsequent variation analysis results.

## 3. Results and Discussion

### 3.1. Phylogenetic Analysis

**Figure 1:**
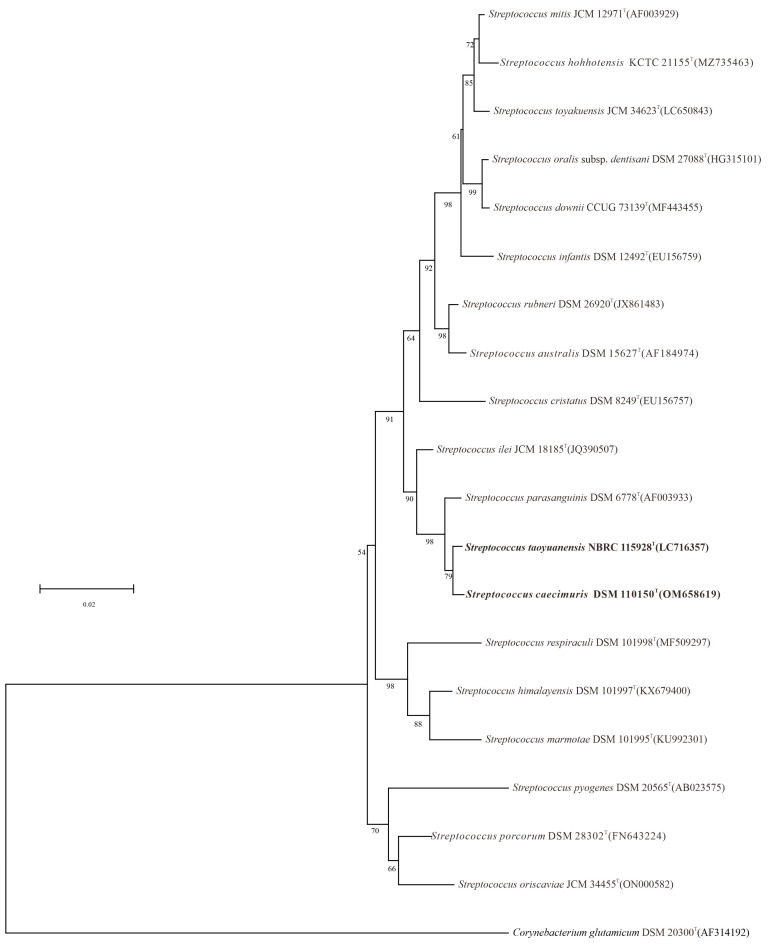
A maximum-likelihood phylogenetic tree was constructed based on 16S rRNA gene sequences, revealing the phylogenetic placement of strains ST2^T^ and CLA-AV-18^T^ among closely related taxa within the genus *Streptococcus*. *Corynebacterium glutamicum* DSM 20300^T^ (AF314192) was selected as the outgroup. Bootstrap values above 50%, derived from 1000 replicate analyses, are displayed at the corresponding nodes. The scale bar corresponds to 0.02 substitutions per nucleotide position.

The results derived from the BLAST sequence alignment analysis clearly indicated a high sequence similarity of 99.6% between the type strain *Streptococcus taoyuanensis* ST2^T^ and *Streptococcus caecimuris* CLA-AV-18^T^, reflecting a high degree of genetic homology at the nucleotide level between these two strains. This high similarity value far exceeds the typical threshold for closely related species within the genus *Streptococcus*, suggesting minimal genetic divergence during their evolution. In addition, the constructed phylogenetic trees based on the relevant conserved gene sequences consistently demonstrated that *S. taoyuanensis* ST2^T^ and *S. caecimuris* CLA-AV-18^T^ clustered together as a well-supported, tight monophyletic clade within the genus *Streptococcus*, with a strong bootstrap value supporting the close phylogenetic relationship between the two strains (Figure 1). This notably high level of sequence similarity identified between *S. taoyuanensis* ST2^T^ and *S. caecimuris* CLA-AV-18^T^, combined with their robust clustering pattern in the phylogenetic analysis, collectively indicates that the two strains share a highly intimate and close evolutionary connection, suggesting they have a common evolutionary ancestor and underwent a similar evolutionary trajectory within the genus *Streptococcus*. The consistent clustering also rules out the possibility of random sequence similarity, further confirming their close taxonomic affiliation.

To further validate the genetic relatedness between *S. taoyuanensis* ST2^T^ and *S. caecimuris* CLAAV18^T^, we analyzed seven conserved housekeeping genes (*atpD*, *gapA*, *gyrB*, *GdhA*, *recA*, *dnaK*, *sdhA*) via the Multilocus Sequence Typing (MLST) database. These genes are essential for basic bacterial cellular functions (e.g., ATP synthesis, glycolysis, DNA replication, metabolism). MLST comparison showed complete concordance in target fragment lengths of all seven genes between the two strains (*atpD*: 41 bp, *gapA*: 39 bp, *gyrB*: 47 bp, *GdhA*: 95 bp, *recA*: 121 bp, *dnaK*: 67 bp, *sdhA*: 33 bp), with no size polymorphisms, variations, deletions, or insertions. This confirms structural integrity of these conserved regions and supports their close genetic relatedness, consistent with 16S rRNA gene analysis.

Concordant fragment lengths provide supplementary evidence for their close relationship, as housekeeping genes are evolutionarily conserved and structural variations are rare in closely related strains. However, identical fragment lengths alone do not confirm complete genetic identity, as small nucleotide polymorphisms (SNPs) or point mutations may exist within sequences, affecting allelic profiles and sequence type (ST) classification. Thus, full-length sequence alignment of the seven housekeeping genes is necessary to determine allelic sequences and assign consistent STs.

Subsequent phylogenetic analysis of these full-length sequences will clarify the precise evolutionary relationship between tao and caecimuris, verify their placement in the same clade, and explore epidemiological linkages (notably, caecimuris from mice and tao from a human bacteremia patient). Combined with 16S rRNA similarity and phylogenetic clustering, this MLST analysis forms cohesive preliminary evidence for their close taxonomic affiliation.

### 3.2. Phylogenomic and Overall Genome Relatedness Indices

**Figure 2:**
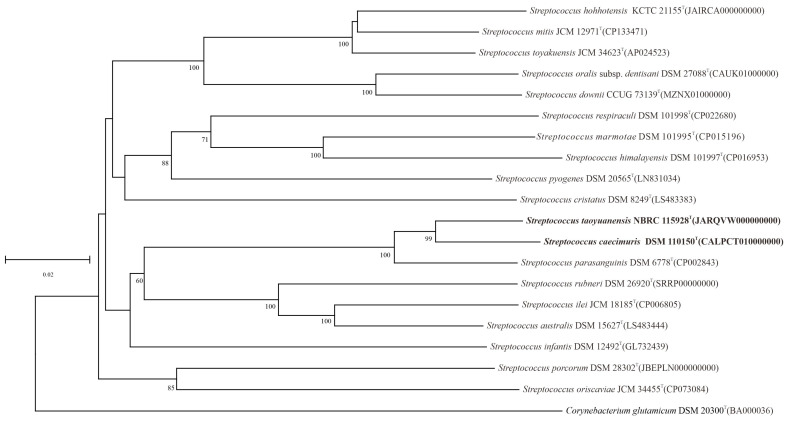
Phylogenomic tree based on whole-genome sequences was constructed using the Type (Strain) Genome Server (TYGS), illustrating the phylogenetic relationships between strains CLA-AV-18^T^, ST2^T^, and related taxa within the genus *Streptococcus*. Branch lengths were calculated according to the GBDP distance formula d5. Support values shown above the branches represent GBDP pseudo-bootstrap probabilities (>50%) based on 100 replicates, with an average branch support of 50.7%. The tree was midpoint-rooted.

As depicted in Figure 2, phylogenetic analysis demonstrated that *S. taoyuanensis* ST2^T^ and *S. caecimuris* CLA-AV-18^T^ formed a robust, well-supported clade within the genus *Streptococcus*. Phylogenetic tree topology further confirmed their distinct separation from other closely related species in the genus. The average amino acid identity (AAI) of 96.8% between these two strains exceeded the 95.5% threshold commonly employed for prokaryotic species circumscription [20]. The ANIb and ANIm values were determined to be 95.6% and 95.8%, respectively, both of which were higher than the 95% species-delimitation cutoff. The digital DNA–DNA hybridization (dDDH) value between the two type strains was 84.6%, far above the 70% threshold for species differentiation. Meanwhile, the genomic DNA G+C contents of *S. taoyuanensis* ST2^T^ and *S. caecimuris* CLA-AV-18^T^ were 42.1% and 42.0%, respectively, further supporting their conspecific status. These genomic indices are widely recognized as the gold standard for prokaryotic taxonomic definition and yield consistent and reliable results in species classification. Collectively, the AAI, ANI, and dDDH values were all markedly higher than the corresponding thresholds for species discrimination, indicative of strong genomic relatedness between the two strains. These convergent genomic metrics confirmed the absence of significant genetic divergence, consistent with their close phylogenetic clustering, and thus provided robust genomic evidence for classifying the two strains as members of the same species.

### 3.3. Pangenomic Analysis and Comparison of Phenotypic and Chemotaxonomic Features

**Figure 3:**
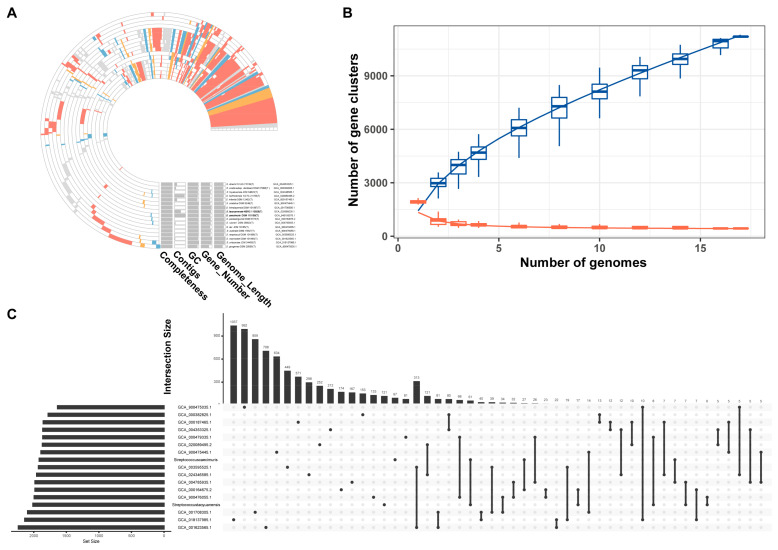
Pangenome analysis was performed across 17 type strains of the genus *Streptococcus*. (**A**) Pangenome overview showing the functional composition of core gene clusters and strain-specific genes within the selected *Streptococcus* genomes. (**B**) Accumulation curve describing the relationship between the number of genomes analyzed and the corresponding count of core gene clusters. The blue line reflects the variation in core gene cluster numbers with an increasing number of genomes included in the pangenome analysis. The orange line generally corresponds to the number of non-core (variable or accessory) gene clusters across different genome sets. (**C**) UpSet plot displaying the distribution of unique genes and shared gene content among the *Streptococcus* strains examined.

**Figure 4:**
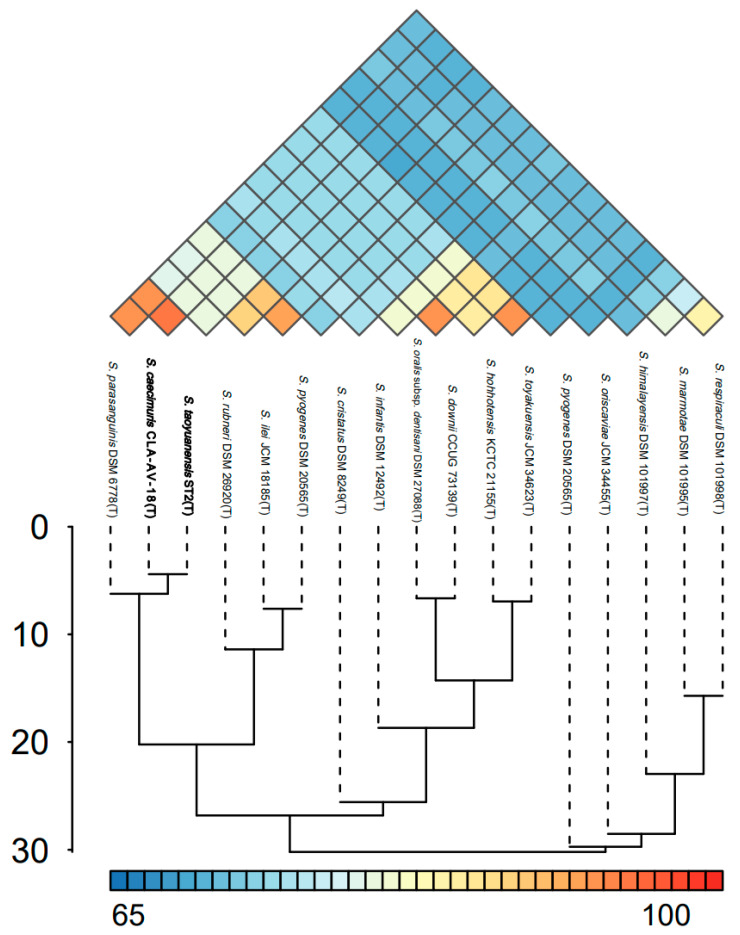
ANI heatmap among the 17 *Streptococcus* type strains.

To investigate the conserved and strain-specific functional characteristics among the two isolates and their related taxa, a pangenome analysis was performed. Based on the IPGA platform, orthologous genes identified from the seventeen selected genomes were divided into core, accessory, and unique gene clusters, as presented in Figure 3. Core gene clusters mainly consist of genes involved in metabolic enzymes, information storage, cellular processes, and signal transduction pathways (Figure 3A), which are indispensable for the fundamental survival and physiological functions of *Streptococcus* strains. Most of the remaining gene clusters were genome-specific unique genes, which may confer unique ecological adaptation and phenotypic traits to each strain. The pan-genome results further revealed the functional conservation and divergence at the genomic level, reflecting the evolutionary adaptation of different strains to diverse ecological niches. The findings from the pangenome analysis were consistent with those from phylogenomic inference, thus supporting the conclusion of close phylogenetic relatedness among these strains via two complementary approaches. In agreement with the above results, ANI strongly supports our hypothesis among 17 *Streptococcus* type strains in this study (Figure 4), with ANI values between the target strains and their affiliated taxa exceeding 95%, a threshold widely recognized for confirming species-level relatedness.

Furthermore, a comprehensive comparison was systematically executed to delineate the distinct phenotypic attributes, along with the biochemical and chemotaxonomic profiles, between the two strains under investigation (Table 1).

Further comparative examination of the phenotypic and chemotaxonomic profiles provided additional support for the above conclusion. Apart from differences in cellobiose utilization (as the sole carbon source) and certain quinone components, *S. taoyuanensis* ST2^T^ and *S. caecimuris* CLA-AV-18^T^ s displayed nearly identical physiological and biochemical characteristics. Both positive for D-glucose, D-fructose, sorbitol, acetyl glucosamine, D-galactose, D-mannose, salicine, maltose, lactose, melibiose, sucrose and D-raffinose, both negative for glycerol, ribose, mannitol, erythritol, L-arabinose, D-arabinose, D-xylose, L-xylose, adonitol, *β*-methyl-xyloside, L-sorbose, rhamnose, dulcitol, α-methyl-D-mannoside, α-Methyl-D-glucoside, inuline, melezitose, glycogene, xylitol, D-turanose, D-lyxose, D-fucose, L-arabitol, D-tagatose, 2-keto-gluconate, L-fucose, D-arabitol, gluconate, amygdalin, inositol, arbutine, cellobiose, trehalose, *β*-gentiobiose and 5-keto-gluconate. The predominant cellular fatty acids of both strains were identified as C_16:0_ C_18:1_*ω*9c, C_18:0_ and C_14:0_ [5,7], with consistent relative proportions, further verifying their high similarity. Additionally, both strains exhibited identical physiological responses to pH and temperature gradients, as well as similar cell morphology under microscopic observation. Therefore, the phenotypic and chemotaxonomic characteristics of *S. taoyuanensis* ST2^T^ and *S. caecimuris* CLA-AV-18^T^ further support this conclusion that *Streptococcus taoyuanensis* (Lee et al., 2024 [10]) is a later heterotypic synonym of *Streptococcus caecimuris* (Afrizal et al., 2022 [8]).

### 3.4. Future Prospects of Microbial Taxonomy

The taxonomic classification of prokaryotes has evolved from the traditional 16S rRNA gene-based framework to a comprehensive, genome-driven system. As demonstrated in the present study, the 16S rRNA gene alone provides limited resolution for closely related species, whereas whole-genome relatedness indices, including average nucleotide identity (ANI), digital DNA–DNA hybridization (dDDH), and average amino acid identity (AAI), serve as robust and reliable benchmarks for accurate species delineation [24]. Future studies on the taxonomy of the genus *Streptococcus* and other bacterial taxa should prioritize phylogenomic and pangenomic analyses to replace or substantially complement conventional single-gene and phenotypic approaches, thereby reducing misidentification and redundant species proposals.

To prevent nomenclatural conflicts and erroneous classification caused by temporal overlaps or asynchronous validation, a centralized, globally coordinated, and open-access data deposition platform is urgently required. This platform should integrate whole-genome sequences, type strain metadata, phenotypic and chemotaxonomic characteristics, isolation source, and formal publication records. Mandatory pre-publication submission and cross-validation of genomic data and strain information on this platform will enable real-time novelty checks, avoid duplicate naming, and ensure consistency with the International Code of Nomenclature of Prokaryotes.

Furthermore, standardized authentication and quality control of type strains must be strengthened across international culture collections. Incorporating genomic similarity thresholds, nomenclatural priority rules, and unified curation protocols into the platform workflow will improve the rigor and traceability of novel species proposals. Collectively, the transition to genome-based taxonomy, supported by a universal data platform and standardized type strain validation, will enhance the stability, accuracy, and interoperability of prokaryotic systematics. This framework will clarify evolutionary relationships within clinically important genera, strengthen microbial diversity research, and support reliable pathogen identification, epidemiological surveillance, and public health management.

## 4. Conclusions

By integrating phylogenetics, comparative genomics, multi-level taxonomic indicators, and phenotypic characterization, this study addresses the taxonomic redundancy within the genus *Streptococcus*. Our findings unequivocally confirm that *Streptococcus taoyuanensis* ST2^T^ and *Streptococcus caecimuris* CLA-AV-18^T^ exhibit extraordinary conservation at the core genome level. Specifically, the complete identity of housekeeping gene fragments revealed by MLST, combined with the high consistency of “gold standard” genomic metrics (AAI, ANI, and dDDH), forms an irrefutable chain of evidence demonstrating a lack of significant evolutionary divergence between the two strains.

In accordance with the principle of priority established by the International Code of Nomenclature of Prokaryotes, and given that the valid publication of *S. caecimuris* predates that of *S. taoyuanensis*, we formally propose the reclassification of *S. taoyuanensis* as a later heterotypic synonym of *S. caecimuris*. This taxonomic revision not only resolves the nomenclatural confusion caused by overlapping publication timelines or asynchronous verification but also provides a precise nomenclatural foundation for future research regarding the clinical infections, ecological distribution, and pathological evolution of this species.

Furthermore, this study underscores the necessity of transitioning from single-gene frameworks to whole-genome-driven taxonomic systems. When characterizing clinically significant genera, the resolution of conventional phenotypic methods and solitary 16S rRNA sequences is often insufficient. Future research should prioritize phylogenomics and pangenome analysis, coupled with globally coordinated open-access platforms, to ensure the rigor, traceability, and long-term stability of new species proposals.

## Figures and Tables

**Table 1:** Differential characteristics between *S. taoyuanensis* ST2^T^ and *S. caecimuris* CLA-AV-18^T^.

Characteristics	*S. caecimuris* CLA-AV-18T^T^	*S. taoyuanensis* ST2^T^
Habitats	A mouse	A patient
Cellobiose	+	−
GC content	42.1%	42.0%

+ positive or present; − negative or absent.

## Data Availability

The original contributions presented in the study are included in the article, further inquiries can be directed to the corresponding authors.

## Abbreviations

The following abbreviations are used in this manuscript:

ANI	Average nucleotide identity.
dDDH	The digital DNA-DNA hybridization
AAI	Average amino acid identity
DSMZ	Deutsche Sammlung von Mikroorganismen und Zellkulturen GmbH
JCM	Japan Collection of Microorganisms
NBRC	Biological Resource Center, NITE
BCRC	Bioresource Collection and Research Center
